# Exploring the Interactions Between Healthcare Professionals and Parents of Children With Feeding Problems: A Qualitative Study

**DOI:** 10.1111/scs.70307

**Published:** 2026-08-21

**Authors:** Jenny Harlid, Anneli Schwarz, Ellen Backman

**Affiliations:** ^1^ Speech and Language Pathology Unit, Institute of Neuroscience and Physiology University of Gothenburg Gothenburg Sweden; ^2^ Department of Research, Education and Innovation Södra Älvsborg Hospital Borås Sweden; ^3^ Department of Social Work Marie Cederschiöld University Stockholm Sweden

**Keywords:** family‐centred care, healthcare professionals, parents, Pediatric Feeding Disorder, professional‐family relations, qualitative research

## Abstract

**Aims and Objectives:**

To describe how healthcare professionals and parents of children with feeding problems experience their interactions.

**Methodological Design:**

A qualitative descriptive design was used, with the Family‐Centred Care (FCC) model as the interpretive framework.

**Research Methods:**

Seven healthcare professionals and six parents participated in individual semi‐structured interviews. Data were analysed using thematic analysis with inductive coding. Trustworthiness was addressed through collaborative analysis, audit trail, reflexivity and triangulation.

**Results:**

The findings resulted in the overarching theme: ‘Navigating the personal and structural effects on healthcare interactions’. Three main themes were developed from the participants' descriptions: ‘Connecting through knowledge’, ‘Why communication matters’ and ‘Finding a way through the system’. The findings show that healthcare interactions are shaped by the level of personal and organizational knowledge in the surrounding environment, the quality of communication and the structure of the healthcare system.

**Study Limitations:**

All participants were Swedish‐speaking from a specific region of Sweden, which limits the applicability of the findings to other cultural or healthcare contexts.

**Conclusion:**

Healthcare interactions are complex and shaped by both personal and systemic factors that influence the experience over time as families move through the care process. Family‐centred care for children with feeding problems should be a continuous goal in healthcare settings. To transition from ‘doing for’ to ‘doing with’ families, healthcare systems must prioritize multidisciplinary coordination, provide education for primary care professionals and address systemic barriers to ensure access and continuity of care. Achieving genuine family‐centred care requires recognizing parents as essential partners on the healthcare team.

## 1 Introduction

Feeding problems in children are often multifactorial and may include food selectivity, oral‐sensorimotor dysfunction and/or limited appetite [1]. These issues can lead to impaired oral intake, defined as ‘inability to consume sufficient food and liquids to meet nutritional and hydration requirements’ [2]. Feeding problems vary in severity and can negatively affect growth, development, general health and family relationships [3]. Approximately, one‐third of typically developing children under the age of five are affected by feeding problems [4], and persistent issues can result in Pediatric Feeding Disorder (PFD) [5], a more complex and long‐lasting condition often associated with medical, nutritional, psychosocial, or feeding skill dysfunctions [2]. A retrospective cohort study [4] found the prevalence of PFD to be 2.7%–4.6% among children under age five. For children with comorbidities, the prevalence was considerably higher: up to 55% in premature children and up to 75% in children with gastrointestinal illness.

Having a child with severe feeding problems can lead to significant emotional distress among parents, which negatively impacts the quality of life for the whole family [6]. The stigma of having a child who eats in a non‐typical way can also discourage parents from seeking help [7]. Several studies report that a lack of awareness of PFD among primary healthcare professionals can result in parents receiving insufficient support and a lack of confidence in the professionals designated to support them [8, 9]. Conversely, receiving appropriate intervention for PFD, such as parent education, reduces the use of ineffective family mealtime strategies compared with no intervention [10]. Multidisciplinary, family‐centred care also helps the healthcare professionals to better support families of children with feeding problems [3].

The starting point of the Family‐Centred Care (FCC) model [11] is that the family's perspective is fundamental to clinical decision‐making. The model emphasizes the need to support the entire family, not just treat the child's problems, as the family serves as the child's primary source of support. While FCC has been internationally recommended as a standard of paediatric healthcare [11], its implementation in universal, community‐based services presents unique challenges related to professional confidence and the nature of the partnership between parents and healthcare professionals [12]. The concept of the ‘feeding relationship’ [13] describes the intricate dynamics between parents and children during mealtimes and includes food selection, eating and regulation of eating behaviours. Creating and sustaining a positive feeding relationship can be difficult in any household. However, when a child has a pediatric feeding disorder, the parent–child feeding dynamic becomes especially fragile and vulnerable to challenges [13, 14]. A better understanding of how daily activities, such as family mealtimes, are organized and modified to meet distinct health care needs is an essential step for healthcare professionals to provide adequate support [15].

Current research primarily focuses on the definitions, prevalence, and interventions for paediatric feeding problems. Few studies investigate the interactions between healthcare professionals and parents of children with feeding problems and how these interactions influence the care process, leaving a clear research gap. Understanding how parents and healthcare professionals communicate and interact is essential to developing family‐centred support. Therefore, this study aimed to explore the experiences of such interactions among both healthcare professionals and parents of children with feeding problems.

## 2 Methods

### 2.1 Research Design

This study uses a qualitative descriptive research design and follows the Consolidated Criteria for Reporting Qualitative Research (COREQ) checklist. Qualitative Description (QD) is a methodological approach that focuses on the ‘who, what and where’ of experiences without deep theorization [16]. This design is particularly well‐suited for clinical research where the goal is to obtain firsthand knowledge that remains close to the participants' own words, ensuring fidelity to their experiences [17]. Given the identified research gap regarding interactions between parents and healthcare professionals, this descriptive approach enabled documentation of these encounters as perceived by the participants.

### 2.2 Interpretative Framework

**FIGURE 1:**
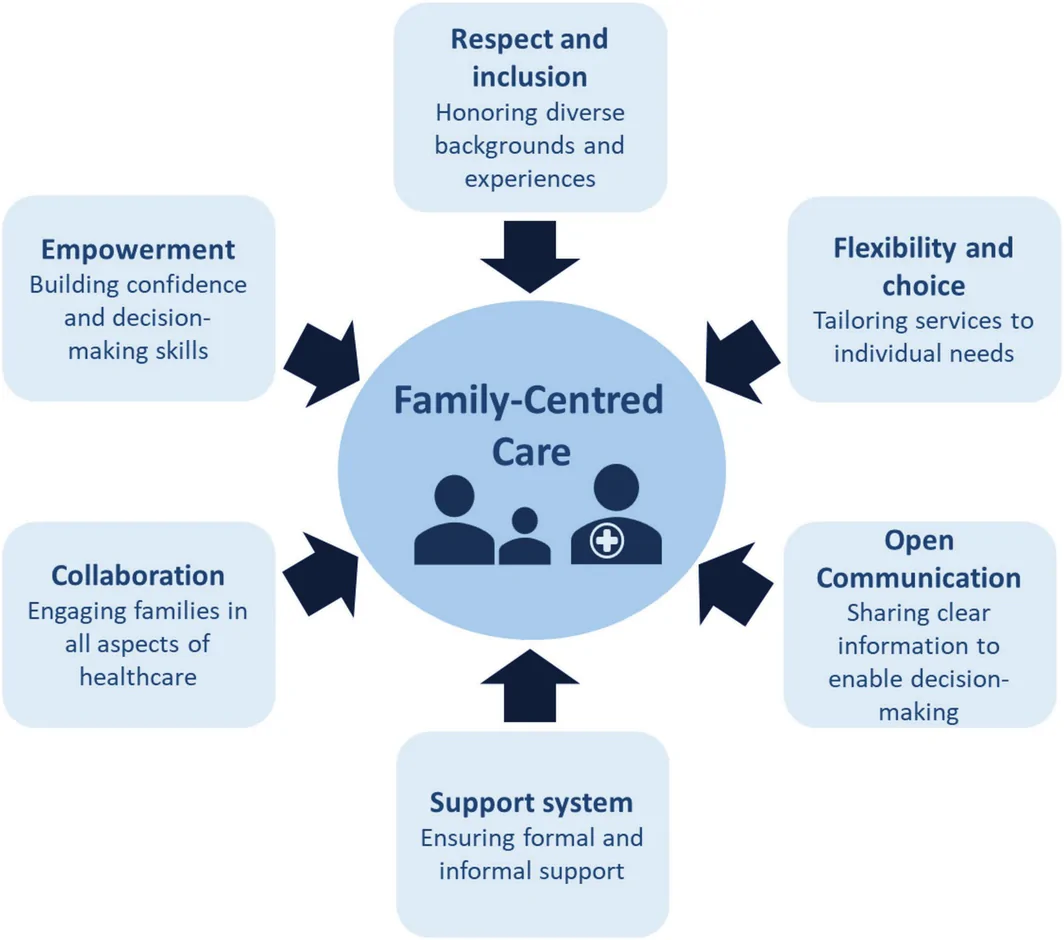
Core principles of the Family‐Centred Care model, based on the Committee on Hospital Care and Institute for Patient‐ and Family‐Centred Care [11].

The straightforward, descriptive approach of QD was complemented by the Family‐Centred Care (FCC) model, which served as the study's interpretive framework to provide analytical depth. This dual approach ensured that the findings remained grounded in the participants' narratives while staying theoretically relevant to current paediatric healthcare practice. As the internationally recognized standard for paediatric healthcare [11], the FCC model enabled evaluation of interactions against principles such as collaboration, empowerment and open communication (Figure 1). These core principles are founded on the partnership between families and healthcare professionals to plan, deliver, and evaluate healthcare [11]. Furthermore, the study's aim aligns with the framework's emphasis on family involvement to build confidence and promote active participation in decision‐making throughout the healthcare journey [11].

### 2.3 Recruitment and Settings

Recruitment was conducted through professional healthcare networks in Region Västra Götaland, Sweden, and by personal invitations. Purposive and snowball sampling strategies were used to capture diverse perspectives, including gender and healthcare professions across various levels of the healthcare system.

All children aged 0–5 living in Sweden are offered services at a Child Health Centre (CHC). The CHC handles all issues related to children's health and development and follows a national programme. Approximately 98%–99% of Swedish children attend the programme and regularly visit the CHC [18]. Children with feeding problems are generally referred from CHC to a Child Clinic, and if the problems persist or worsen, a further referral is made to a Paediatric Nutrition Team, which may include specialists such as dietitians, nurses, speech‐language pathologists, physicians, psychologists, physiotherapists and occupational therapists.

### 2.4 Participants

Following informed, verbal consent, seven healthcare professionals and six parents participated in individual semi‐structured interviews between January 31st and May 9th, 2025 (Table 1). Participants included healthcare professionals working with paediatric feeding problems and parents of children aged 9 months–7 years with significant feeding problems as indicated by a documented diagnosis of PFD or a score of 9 or more on the Behavioural Paediatric Feeding Assessment Scale (BPFAS) [19]. The children's age range was determined by selecting the age categories included in BPFAS. All participants were mandated to be Swedish‐speaking. Participants did not need to have an existing patient‐healthcare provider relationship. None of the participants had a clinical or therapeutic relationship with any of the authors, and the authors were not involved in the participants' clinical care, assessment or treatment at any stage of the study. However, as recruitment was partly conducted through professional networks, some healthcare professional participants were known to the first author as colleagues.

**TABLE 1:** Characteristics of the study participants.

Characteristic	Healthcare professionals (*n* = 7)	Parents (*n* = 6)
Age: *n* (%)		
30–39 years	2 (29)	4 (66)
40–49 years	2 (29)	2 (33)
50–59 years	3 (43)	—
Gender: *n* (%)		
Male	1 (14)	1 (17)
Female	6 (86)	5 (83)
Profession: *n* (%)		
Dietician	1 (14)	N/A
Physician	1 (14)	N/A
Nurse	3 (43)	N/A
Speech‐language pathologist	2 (29)	N/A
Professional experience related to children with feeding problems: *n* (%)		
1–5 years	1 (14)	N/A
6–10 years	5 (72)	N/A
> 20 years	1 (14)	N/A
Level of care: *n* (%)		
Primary care (Child health centre)	2 (29)	N/A
Secondary care (Child clinic)	1 (14)	N/A
Tertiary care (Paediatric nutrition team)	4 (57)	N/A

Abbreviation: N/A, not applicable.

The children of the participating parents included three girls and three boys, aged 1–6 years, with a mean age of 5.1 years.

### 2.5 Data Collection

All interviews were conducted individually by the first author via video calls with a mean length of 25 min (range: 15–37 min). Interview guides were used (Tables S1 and S2), comprising open‐ended questions focused on family‐centred care aspects (such as shared understanding and decision‐making) and on past and present personal experiences with healthcare interactions. An example of an interview question directed at healthcare professionals is ‘How do you involve parents in the care of their child's feeding problems’? The initial interview guide was piloted through a test interview with a healthcare professional and subsequently refined to remove closed‐ended questions and to include more FCC‐related questions. The questions were also adjusted as necessary during the interviews. The collected data consisted of video recordings from interviews. These recordings were transcribed verbatim by the first author, and the transcripts were the empirical basis for analysis.

Data sufficiency was guided by the concept of information power, which holds that sample size depends on the study's aim, sample specificity, quality of dialogue and analysis strategy [20]. In this study, information power was achieved through several factors. First, the study's aim was specific, focusing on interactions within the specialized area of paediatric feeding problems. Second, the participants were purposively selected to ensure they possessed firsthand, relevant experience and to represent diverse healthcare professions and parents of children with documented feeding problems. Finally, the use of QD as a research design supported the adequacy of the sample size (*n* = 13) by staying close to participants' own words to capture the ‘who, what, and where’ of their experiences.

### 2.6 Data Analysis

The first author, a speech‐language pathologist with extensive clinical experience working with children with feeding problems and their parents, was responsible for the data analysis using thematic analysis (TA) as described by Braun and Clarke [21]: (1) Familiarization with the data through verbatim transcription and repeated reading. (2) Systematic inductive coding of the entire dataset. (3) Generating initial themes and subthemes. (4) Reviewing themes against the coded extracts and the entire dataset. (5) Defining and naming themes and subthemes. (6) Further refining themes until the final themes and subthemes were established. Table 2 illustrates this analytical process, providing a direct link from raw interview extracts to the final themes.

**TABLE 2:** Overview of the analytical process from interview extract to final theme.

Interview extract	Code	Subtheme	Theme
‘It needs to be talked about more, and more information needs to be given to both doctors and nurses who work with children that…this problem exists’ (Parent 1)	Need for increased knowledge	The value of knowledge and comprehension	Connecting through knowledge
‘We didn't reach a mutual understanding, and we couldn't agree, and eventually the contact just faded away because we couldn't connect’ (Healthcare professional 2)	Hard to reach a shared understanding	The dynamics of communication and building trust	Why communication matters

To support a transparent approach and mitigate potential interpretive bias, particularly given the first author's clinical background as a speech‐language pathologist, several measures were implemented. First, a reflexive journal was kept throughout the process to document initial impressions and challenge personal assumptions and potential biases, thereby becoming aware of the influence of personal experience on data interpretation. Second, co‐authors were consulted at every step of the analysis, from defining and reviewing the codes, subthemes and themes to ensure the credibility of the findings. Consensus among all authors was high throughout the analysis, and only a few modifications were made following the discussions. Finally, transparency was supported by a detailed audit trail and constant comparisons between emerging themes and the original transcripts.

### 2.7 Trustworthiness and Reflexivity

Ensuring trustworthiness requires attention to credibility, dependability, confirmability and transferability [22]. Credibility refers to the accuracy of the interpretation of the participants' meaning [22] and was maintained through systematic data analysis. Collaborative analysis among the authors minimized individual bias and strengthened credibility. Dependability and confirmability were supported by an audit trail that documented research decisions and reflections throughout the process. The authors maintained a reflexive stance, acknowledging how personal background, assumptions, and professional experience might influence data interpretation. Transferability was enhanced through detailed documentation of the research steps and transparent reporting of data collection and analysis. Triangulation, which refers to using multiple methods or data sources to investigate the research question and enhance confidence in the findings [21], was achieved by analysing perspectives from both healthcare professionals and parents, thereby broadening understanding of the phenomenon under study.

The first author, a speech‐language pathologist with extensive clinical experience in pediatric feeding disorders, conducted all interviews and led the analysis. This dual role as both ‘expert’ and ‘researcher’ influenced the study's trustworthiness in several ways. Regarding data generation, the first author's clinical background facilitated trust with both parents and healthcare professionals through a shared professional language and an understanding of the clinical complexities of PFD. This enabled the use of nuanced follow‐up questions, allowing participants to delve deeper into their experiences, a process supported by literature on the benefits of insider research in clinical settings [23]. However, the researcher remained vigilant throughout the interviews to ensure this expertise did not lead to steering participants or making assumptions about their intended meanings. The potential influence of the professional relationship between the interviewer and some participants was addressed through reflexive journaling and by involving co‐authors in the thematic analysis to ensure the findings remained grounded in the data.

### 2.8 Ethical Considerations

The study was conducted in accordance with the principles of the Declaration of Helsinki (World Medical Association, 2024), ensuring participants' rights to informed consent, confidentiality and voluntary participation. Ethical approval was obtained from the Swedish Ethical Review Authority (250107/2024‐07598‐01). The data were pseudonymized and related only to a given code to protect participants' privacy. The material was available only to the researchers involved in the study. Special care was taken to maintain the pseudonymity of the participants' descriptive answers when reporting the findings.

## 3 Results

**FIGURE 2:**
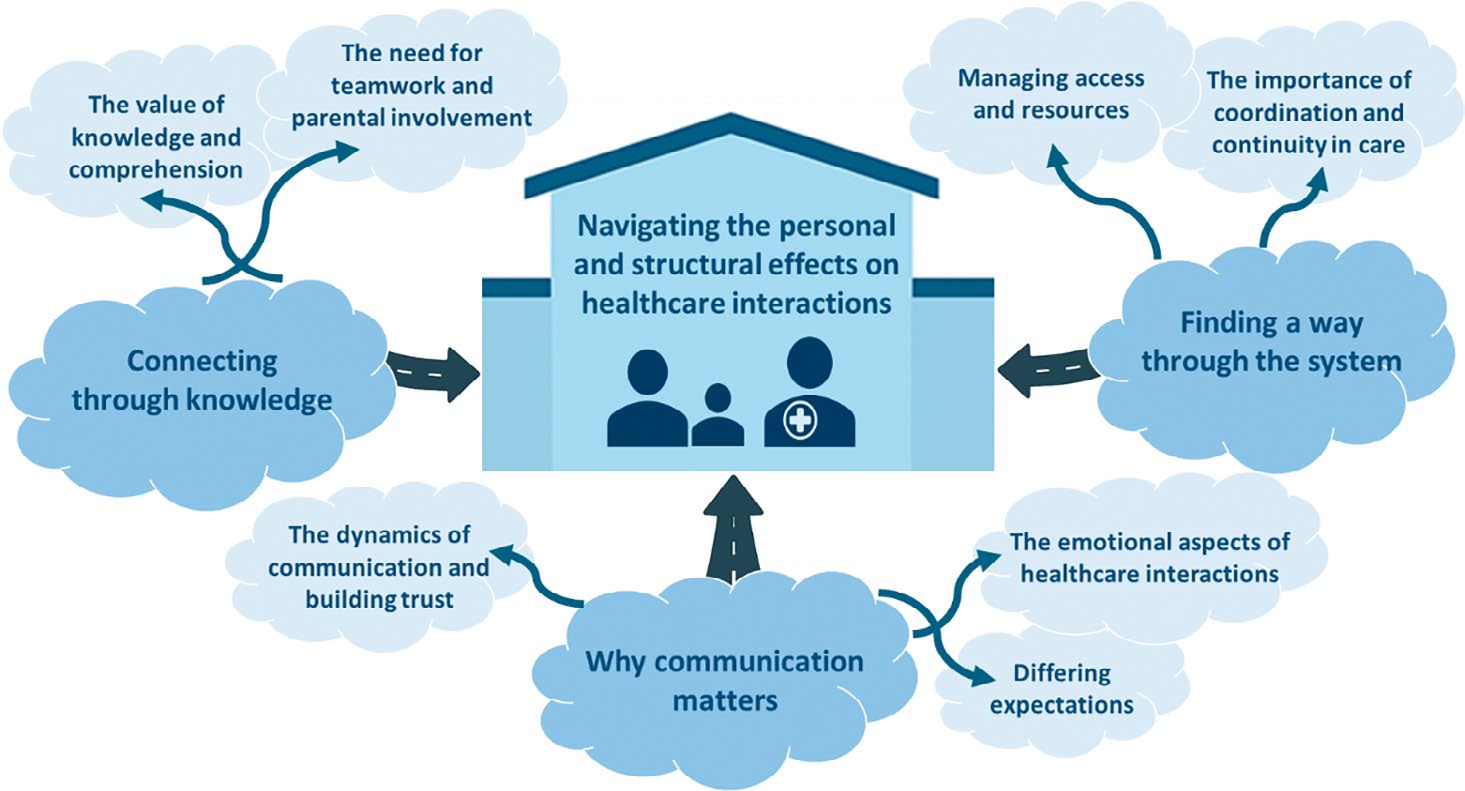
Overview of the overarching theme, main themes and subthemes.

At the centre of both parents' and healthcare professionals' stories of healthcare interactions were the interconnected experiences related to knowledge, communication and access to healthcare when managing paediatric feeding problems. When compiling the results, the analysis focused on the ‘who, what, and where’ in the participants' narratives, using the qualitative description approach. The experiences were summarized under the overarching theme, ‘Navigating the personal and structural effects on healthcare interactions’, illustrating that healthcare interactions are not simple and straightforward but rather a complex, time‐consuming journey to find and maintain appropriate care (Figure 2).

Complexity arises at the intersection of personal factors, such as knowledge, expectations and emotions, with practical and organizational factors related to access and care coordination. The following quote from a parent depicts this challenge: ‘How do parents and healthcare professionals find each other in a way that…that it doesn't have to take so long to pinpoint this problem’? (Parent 5).

The overarching theme branched into three main themes and seven subthemes, which are addressed in turn. Participants are referred to as HP (Healthcare Professional) and P (Parent), each followed by a number.

### 3.1 Connecting Through Knowledge

The participants' views on knowledge include individual knowledge and skills, as well as shared knowledge developed through teamwork and parental involvement. Experience and understanding are crucial to parents' and healthcare professionals' ability to connect and collaborate effectively. However, many parents struggle to be acknowledged and actively involved in interactions.

In the subtheme, ‘The value of knowledge and comprehension’, participants share insights into how both formal education and experience‐based knowledge of feeding problems in children influence healthcare interactions. One healthcare professional noted how experience can alter the way one conveys information to parents:

Having more experience also means that one can speak with greater confidence about things like ‘this is how we usually do it’, or ‘this has been my experience’, and that there is also some substance to it, that it's not just something I made up or ‘I read this somewhere’, but that there are actually some truths that I bring. (HP2)

Parents also emphasize the importance of meeting healthcare professionals with more in‐depth knowledge and understanding, for example: ‘When we've had the chance to meet… staff who were more specialized in this area, then they've been able to grasp what we're saying in a different way’ (P2). This experience is supported by several reports indicating that interventions at the primary level of care were perceived as inadequate by both healthcare professionals and parents. For instance: ‘I also feel that the Child Health Center has said things like, ‘Yeah, just keep going’, and we are doing that, but maybe we haven't received very much practical advice’ (P3). Correspondingly, one healthcare professional expressed a need for professional development: ‘… to gain a better understanding of what feeding problems are and which children are affected, especially for me here at the Child Health Center’ (HP5).

The second subtheme, ‘The need for teamwork and parental involvement’, reflects the importance of a multidisciplinary approach and the value of parents feeling involved and viewed as part of the team.

Both parents and healthcare professionals highlight the benefits of team meetings. One healthcare professional described that:

Having team meetings really reflects the principle that one plus one equals three. You bring your own knowledge, the other person brings theirs, and when you combine them, the result is even better than if each had worked separately. (HP1)

Another aspect of this subtheme is the relevance of including parents' perspectives in interactions. For example, one healthcare professional expressed the role of parents as ‘For me, they are a very important part because they are usually the ones who know their children, know how they function, know how to motivate the children in different ways, and what they like’ (HP3). Other healthcare professionals mentioned the importance of considering the parents' situations by asking questions and encouraging them to share their everyday experiences. This family‐centred approach is further reflected in healthcare professionals' statements indicating that they aim to adapt support to each family's situation and needs.

When it comes to parental involvement, the data reveal a range of experiences, from feeling disconnected to feeling satisfied with the level of inclusion. As shown across many of the interviews, the same participant can feel both satisfied and dissatisfied, depending on the healthcare interaction being referenced. One parent expressed feeling overlooked during the initial stage of seeking support at the CHC: ‘Then you're standing there and just…a really long time has passed, and we haven't heard anything, what's going on? What's the plan? And then you feel annoying and pushy if you call and […] you end up feeling forgotten’ (P6). However, the same parent also reported feeling well‐informed during later encounters with healthcare professionals when the interactions occurred in a more specialized healthcare setting: ‘But I've still felt that they've been very good at explaining what the plan is, we're thinking this is the plan because we want to try this and we're planning to do that’, and it makes you feel involved’ (P6).

### 3.2 ‘Why Communication Matters’

This second theme explores the importance of communicating clearly and establishing trust, as well as experiences related to responsiveness and expectations in interactions. Participants repeatedly emphasize that honesty and transparency form the foundation for successful interactions. Both healthcare professionals and parents stress the importance of open communication to avoid misunderstandings and create realistic expectations.

In the first subtheme, ‘The dynamics of communication and building trust’, several accounts highlight that attentiveness and responsiveness from healthcare professionals lead to positive interactions. Both parties report that many encounters felt positive, especially when shared understanding and trust were established, as described by this healthcare professional:

Meetings where there is good communication and a good relationship between everyone, where you can trust each other and… well, when the family also feels involved in the conversations. Then I feel that things work much better, that you reach some kind of shared conclusion that everyone agrees on. Those are good meetings, I think. (HP3)

However, parents also report facing communicative barriers like not being heard when describing the child's feeding problems or healthcare professionals focusing on the wrong issue. These experiences highlight the importance of parents being very clear in describing food‐related challenges for healthcare professionals to fully grasp the severity of the situation. One parent recalled: ‘I felt like you kind of had to be rude for them to listen to what I was saying […] At some point, I've had to be that rude, like, saying I'm not leaving until I get to speak to a doctor’ (P6). This sentiment was mirrored among healthcare professionals, who acknowledged that parents might feel that they are not being heard or taken seriously when advocating for their child.

The findings also show that when parents and healthcare professionals disagree, parents may experience accusations or distrust from healthcare professionals, which in turn can lead to a lack of trust. One parent said, ‘Unfortunately, I didn't feel I could be honest about how much formula he drinks, because then you get criticized for offering formula, but that's been the only thing he's been willing to eat’ (P1).

The second subtheme, ‘The emotional aspects of healthcare interactions’, highlights key topics, including parents' experiences of stress and worry, healthcare professionals' feelings about their roles and how these factors influence interactions. Parents report feeling stressed by healthcare professionals' perceptions of what and how much their child should eat. One parent commented this:

In the beginning, you kind of felt this pressure to make sure she got enough food and all that. I already felt that at the Child Health Center, with the supplements and everything, she [Child Health Nurse] really pressured me, like, ‘you have to stop breastfeeding, you have to give supplements’. (P4)

Healthcare professionals, on the other hand, express ambivalence toward the advice they give parents, indicating doubt about their professional role. For example: ‘If they're breastfeeding, it's so hard to say that maybe they should cut back or stop breastfeeding to get started with solid foods… I find it really difficult sometimes that they have to remove something we generally encourage’ (HP7). This sentiment was further reflected in the parents' accounts, expressing that both parties could feel uncertain about the core of the child's feeding problem and how to address it.

Healthcare professionals' accounts also show awareness of how emotionally charged meals can be for families and of the expectation that healthcare encounters will involve strong reactions from parents. One healthcare professional stated: ‘There's just so much emotion tied to food when it comes to parents, I think.’ (HP7), while another noted that: ‘A meeting can involve lots of emotions and a great deal of worry. It's not at all uncommon for there to be tears during the meetings’ (HP4).

The last subtheme, ‘Differing expectations’, sees both parents and healthcare professionals share examples of encounters in which their expectations align, but more often they do not. Reasons for this misalignment vary: cultural differences, disparate views of the child's problems, or expectations that the other party felt unable to meet.

The accounts show that many parents actively seek help for their child's feeding problems, and that healthcare professionals are aware of parents' expectations for support. However, opinions on what the support should include vary between the two groups, and both parents and healthcare professionals express that meeting the other's expectations can be difficult. One healthcare professional explained: ‘Some, I think, want help now, and it's difficult to explain that this is a process that sometimes takes several years’ (HP3).

The accounts indicate that parents are generally motivated to meet healthcare professionals' expectations by following the advice and recommendations they receive. Still, they can sometimes struggle to follow through. One parent described this challenge:

What hasn't worked is when she needs to take something for constipation. She doesn't want to eat regular food, so it's not easy to get her to take medication and things like that. So, at times, some things have felt almost unreasonable. (P5)

Healthcare professionals, from their perspective, also express frustration when parents are unable to give their child medication. One healthcare professional described it like this:

Then it feels like we can't move forward when we're unable to motivate the parents and the child to take their medication, because in that case, we can't help them. And that's also frustrating, I think’. (HP3)

### 3.3 ‘Finding a Way Through the System’

This third theme covers the practical aspects of engaging with the healthcare system, focusing on participants' perspectives on access to healthcare and the complexities of coordinating a continuous treatment plan.

Within the first subtheme, ‘Managing access and resources’, participants express that receiving appropriate care can be a lengthy process. They highlight both structural barriers within the healthcare system, such as strict referral guidelines and ineffective patient flows, and external factors that delay appointments, including illnesses, resulting in missed or delayed support.

Both parents and healthcare professionals emphasize the importance of identifying feeding problems in children early to prevent or reduce more serious issues. One healthcare professional said: ‘I think it's so important. Their food journey right from the start, that's where you might be able to do more, so that it doesn't develop into a feeding problem later on, maybe’ (HP5). However, this shared understanding was contrasted with a feeling among parents that they ‘had to push’ to receive adequate care. One parent put it this way: ‘I don't think it should have to go that far that I, as a parent, have to fight for my child's right to receive proper care’ (P1). Parents note that support from healthcare can feel both inadequate and sufficient depending on the situation. The same parent might express both views, highlighting that navigating access to healthcare and its resources is an ambivalent experience.

The experiences of long waiting times and delays in accessing care play a significant role in this subtheme, as these create barriers to timely interventions. One parent stated: ‘There's been a parent education program that we'd been hearing about for a year, but we only got to start attending it a few weeks ago, because the waiting list was so long’ (P3).

The final subtheme, ‘The importance of coordination and continuity in care’, addresses issues of coordination across the healthcare system. It examines how participants perceive different aspects of treatment planning and how experiences can vary depending on who they interact with. The accounts indicate that not having access to a multidisciplinary team, but instead having separate contact with many healthcare professionals, is viewed as a disadvantage, as healthcare units become isolated, with limited information sharing and coordination. These isolated healthcare contacts are not only time‐consuming but also risk placing responsibility for overall coordination on the parents.

Healthcare professionals also recognize this gap of coordination: ‘They're sometimes waiting for test results that we think they've already received […] or they've been to examinations where the care contacts don't… follow up on each other, but instead run like parallel tracks, with no communication in between’ (HP6). Both parties believe that maintaining contact with the same healthcare professionals over time helps build a stronger foundation for intervention planning that fits the needs of the family and the individual child. One parent said this:

Since we've had the same doctor the whole time. And also [name of speech‐language pathologist] […] and they've gotten to know our daughter and us and understand what challenges she has in terms of her personality, and that has made us feel […] that they've come to understand more and more what things are like from the inside. (P4)

Parents also described the consequences of poor coordination and a lack of information as challenging for the family, as this parent noted: ‘I probably would have wanted, from the beginning, a clearer plan for what you're supposed to do in a situation like this. But we felt a bit left on our own, just waiting for something without knowing what we were waiting for or how long we would have to wait’ (P2).

## 4 Discussion

This study aimed to explore how healthcare professionals and parents of children with feeding problems perceive their interactions, using the FCC model as an interpretative framework. The overall finding is that perceptions of interactions within healthcare are influenced by both personal and structural factors and that the experience may change over the course of the family's journey through the healthcare system. It is evident from the participants' accounts that FCC is an aspirational goal, but that it can be hard to fully achieve.

The first main finding is that both healthcare professionals and parents find it essential to collaborate to increase knowledge and understanding, but that this can prove challenging depending on who you interact with. The second main finding shows that open communication and feeling heard are essential to facilitate positive interactions and adequate support. The last main finding focuses on how interactions are affected by the structure of the healthcare system, such as accessibility, waiting times and coordinated appointments. Together, the findings align with previous research on family‐centred care, which emphasizes the importance of family involvement [11].

In the first main finding, a strong knowledge foundation emerges as a vital aspect in the interviews, and parents clearly feel more connected to healthcare professionals working in specialized settings with greater experience. Prior reports [9, 24] have noted that a lack of awareness among primary healthcare professionals regarding paediatric feeding problems can result in parents not receiving the support they need. This study shows similar results and also suggests that parents may be more open‐minded when they know they are meeting with a specialist team, and therefore more likely to connect during those interactions. It also shows that healthcare professionals at the primary care level tend to express greater doubt about their knowledge and a desire to improve their understanding of paediatric feeding problems. These results are consistent with previous research [25], which highlights a lack of awareness of PFD among referring professionals and underscores the need for improved training and systemic changes. In this study, parents often report feeling overlooked at the primary level, aligning with the scoping review by Ridgway et al. [12], which emphasizes that delivering genuine family‐centred care in community‐based health services requires addressing systemic barriers and enhancing the specific skills needed to work effectively with families. The combined findings of this study suggest that, without sufficient knowledge, healthcare professionals may lack the confidence to refer families to appropriate support, thereby weakening the FCC model's flexibility and choice principle.

The second main finding is the importance of building trust through open communication to manage differing expectations. Both parties found it difficult at times to meet each other's expectations regarding the type of support that would best suit the family. Parents' reports of feeling stressed by healthcare professionals' opinions about the child's eating and of struggling to feel acknowledged and heard create barriers to communication. This, in turn, leads to a lack of trust that weakens the collaborative relationship recommended by the FCC model. This second main finding is supported by McCarthy and Guerin [26] and Dadich et al. [27], who also concluded that parents and healthcare professionals struggle to find common ground when beliefs, feelings or actions are unclear due to differing perspectives. In line with the FCC model, participants noted that open communication and parental empowerment were vital to achieving positive outcomes in interactions. This notion is consistent with previous research showing that incorporating strategies such as listening, shared decision‐making and ensuring support benefits interactions between clinicians and caregivers [28]. Parents' accounts show that they feel included simply by receiving a treatment plan and that healthcare professionals believe they include parents by sharing one. However, the accounts rarely mention engaging the parents in creating the plan, suggesting an unawareness of how to move beyond ‘doing to’ or ‘doing for’ families toward ‘doing with’, as also described by Britt et al. [29].

The third main finding is that access to continuous and coordinated care is key for parents to feel that their concerns regarding the child are taken seriously and is a substantial source of frustration in interactions between parents and healthcare professionals when it does not work. Parents report that long waiting times, without knowing what comes next, put an extra strain on the family. This finding aligns with previous research showing that healthcare services can be perceived as disorganized and unsatisfactory when families are sent between different caregivers and may be recommended unrealistic treatment options or no treatment at all [25].

In this study, receiving appropriate support was reported to be challenging for many parents, leading them to often feel pushed into an advocacy role against the system rather than functioning as respected partners within it. Consistent with the FCC model, participants noted that a functioning support system was essential to meeting families' needs.

The study's three main findings clearly connect to several of the FCC model's core principles. In line with the principles of flexibility and choice and inclusion, the first main finding shows that healthcare professionals aim to tailor the care they provide to what works best for the family, and that this approach is most effective when the family and healthcare professionals build a strong foundation of knowledge and shared understanding. The second main finding confirms that when the principles of open communication and empowerment are followed, parents feel heard and taken seriously. When these principles are not followed, parents may perceive healthcare professionals as unresponsive, leading to frustrating interactions. Lastly, the third main finding shows that maintaining continuous contact with healthcare professionals is important to parents, but obtaining it can be time‐consuming due to structural challenges such as poor coordination and limited access, making the support system a difficult FCC principle to adhere to.

### 4.1 Clinical Implications

In terms of improving clinical practice, the findings indicate a clear need to increase knowledge and awareness of paediatric feeding problems and FCC among healthcare professionals, especially at the primary care level. The findings showed that healthcare professionals with a deeper understanding of feeding problems in children can grasp the issue from a different perspective, promoting mutual respect, inclusion and collaboration with parents. A suggestion to increase healthcare professionals' awareness of feeding problems in children is to provide routine education on how to identify these children early and ensure that they are referred to the appropriate services. Lastly, the findings on access and coordination show that structural factors often obstruct effective FCC implementation and that for healthcare interactions to be genuinely helpful, support must be provided early in sufficient quantity and involve the family.

### 4.2 Limitations and Suggestions for Future Research

A strength of this study was the inclusion of both parents and healthcare professionals, rather than interviewing only one group. This dual perspective, together with the qualitative descriptive design that stays close to the participants' own words, deepens understanding of what happens during interactions. Although participants were predominantly female, the study included male participants in both groups, which was considered a meaningful contribution. However, several limitations need to be considered. Although small sample sizes are common in qualitative research [22], including only seven healthcare professionals and six parents may result in findings that do not fully capture all relevant aspects of healthcare interactions. Despite the small sample size, the information power derived from the specificity of participants' experiences and the specialized clinical area of paediatric feeding problems was deemed sufficient to address the study aim. Additionally, all participants were Swedish‐speaking from a specific region with approximately 1.7 million inhabitants, in both urban and rural areas. This might limit the applicability of the findings to other cultural or healthcare contexts. Furthermore, the use of individual semi‐structured interviews presents data that consists of reconstructed experiences and narratives. While these narratives provide deep insight into what participants think and feel about their interactions, they may not always align with actual clinical practice. Consequently, the findings should be interpreted as the participants' subjective perceptions of their interactions rather than as direct observations of encounters. To address this, future studies could consider observations of healthcare interactions as a valuable data‐collection method to compare reported experiences with real‐time practice. Another limitation is that the perspectives of other relevant professionals working with children with feeding problems, such as counsellors, clinical psychologists and occupational therapists, were not included, which could have provided additional valuable insights.

In the future, a larger‐scale qualitative study could be conducted to explore the experiences of a more diverse sample of healthcare professionals and parents, including those from other professions and diverse geographic and cultural backgrounds.

## 5 Conclusion

The findings of this study show that healthcare interactions for paediatric feeding problems are a complex relational journey shaped by personal and structural factors that are not easily separated. Parents and healthcare professionals are left to balance these factors throughout their encounters, making the navigation of paediatric feeding problems far more than a medical challenge. While these findings are rooted in a specific Swedish regional context and the reconstructed experiences of 13 participants, they offer valuable insights for moving toward genuine family‐centred care.

To transition from ‘doing for’ families to ‘doing with’ them, three key implications are identified.

Clinical practice: Healthcare systems should prioritize multidisciplinary coordination to relieve parents of the burden of self‐advocacy and to ensure continuity of care.

Education: The findings indicate a need for routine training for primary care professionals to bridge the identified knowledge gap and enable earlier, more confident referrals.

Research: Future studies should include a more diverse range of professional and cultural groups to test the transferability of these findings. Observational methods could also be used to compare reported interactions with real‐time practice.

Finally, achieving FCC in the care of children with feeding problems requires a systematic commitment to viewing parents not as recipients of care but as essential partners on the healthcare team.

## Author Contributions

J.H. conducted the interviews, performed the data analysis, interpreted the findings and drafted the manuscript. E.B. and A.S. contributed to the study conception and design, participated in the data analysis and interpretation of the findings, critically revised the manuscript and supervised the research process. All authors read and approved the final manuscript and meet the ICMJE criteria for authorship.

## Funding

This work was supported by grants from the Department of Research, Education and Innovation, Södra Älvsborg Hospital, Borås, Region Västra Götaland, Sweden.

## Ethics Statement

Ethical approval for this study was granted by the Swedish Ethical Review Authority (250107/2024‐07598‐01). The research was conducted in accordance with the Declaration of Helsinki, upholding participants' rights to confidentiality and voluntary participation.

## Consent

Informed consent was obtained from all participants involved in the study.

## Conflicts of Interest

The authors declare no conflicts of interest.

## Supporting information


**Table S1:** Interview guide for parents.
**Table S2:** Interview guide for healthcare professionals.

## Data Availability

The data are not publicly available due to ethical restrictions.
