# Supplementary material for: Exploring the Interactions Between Healthcare Professionals and Parents of Children With Feeding Problems: A Qualitative Study
Source: Scand J Caring Sci. 2026 Aug 21;40(3):e70307. doi: 10.1111/scs.70307 (PMC13498768; doi:10.1111/scs.70307)
Supplement: Supplementary file 1 [file SCS-40-0-s001.docx]

**Supplementary material**

**Supplementary Table 1:** Interview guide for parents
**Supplementary Table 2:** Interview guide for healthcare professionals

**Supplementary Table 1:** Interview guide for parents

**Questions for Parents**

1) First, could you describe how your contact with healthcare started regarding your child´s feeding problem?
   a) How have the meetings with healthcare been since then?
2) What were your expectations when first seeking help for your child´s feeding problems?
   a) How have your expectations possibly changed over time?
   b) What expectations do you believe healthcare professionals have had of you as a parent during your meetings?
3) If you think back to your healthcare meetings with healthcare professionals, can you tell me about a meeting that felt very good?
   a) What else do you think makes meetings with healthcare regarding your child´s eating difficulties good?
   b) Can you tell me about other positive healthcare meetings you have experienced?
4) Can you tell me about a meeting with healthcare professionals that was particularly difficult or challenging?
   a) What else makes meetings with healthcare regarding your child´s feeding problems difficult?
   b) Can you tell me about other challenges you have experienced in healthcare meetings?
5) From your perspective, what have healthcare professionals done to understand your child´s feeding problems and your challenges as a parent?
6) How have healthcare professionals involved you in making decisions about your child´s eating difficulties?
   a) How did it feel when you made important decisions in those situations?
7) Thinking back, how do you feel healthcare professionals have treated you regarding your child´s feeding problems?
   a) What do you think about the advice and interventions you have received for your child´s feeding problems?
8) What would you have wanted to change in your meetings with healthcare professionals regarding your child´s feeding problems?
9) Is there anything else you´d like to share or anything I´ve overlooked asking about your experience with healthcare professionals regarding your child´s feeding problems?
10) Do you have any questions for me?
11) Would you like to receive the research article when it is ready?
12) Would it be okay if I contacted you again if I have any questions about the interview?

**Supplementary Table 2:** Interview guide for healthcare professionals

**Questions for Healthcare professionals**

1) First, could you share a bit about what your work involves concerning children with feeding problems?
2) What education and professional experience do you have regarding children with feeding problems?
   a) In what way does your knowledge feel sufficient or insufficient for your work with children with feeding problems?
3) If you think back to your meetings with parents, can you tell me about a meeting that felt very good?
   a) Can you tell me more about what makes meetings with parents of children with feeding problems feel like good meetings?
   b) Can you share more positive meetings you have experienced?
4) Can you tell me about a meeting with parents that was particularly difficult or challenging?
   a) Can you tell me more about what makes meetings with parents of children with feeding problems feel difficult or challenging?
5) What do you experience as the biggest challenge in your meetings with parents of children with feeding problems?
   a) What else can be challenging?
6) What expectations do you have of the parents in the meeting?
   a) And if you think back, how have your expectations possibly changed over time?
   b) What do you think the parents´ expectations are of you?
7) How do you involve parents in the care of their children´s feeding problems?
8) What would you want to change regarding your meetings with parents of children with feeding problems?
9) Is there anything else you´d like to share, or anything I may have overlooked in asking about your experience meeting parents of children with feeding problems?
10) Do you have any questions for me?
11) Would you like to receive the research article when it is ready?
12) Would it be okay if I contacted you again if I have any questions about the interview?
